# A Systematic Review of Literature: TNF‐α Blockers and JAK Inhibitors for the Treatment of Stevens‐Johnson Syndrome, Toxic Epidermal Necrolysis, and Severe Forms of Erythema Multiforme

**DOI:** 10.1002/hsr2.72668

**Published:** 2026-06-19

**Authors:** Sadaf Salehi, Pooneh Torabi, Mahsa Abbaspour, Nima Hajizadeh, Sara Sadeghi, Alireza Jafarzadeh, Azadeh Goodarzi

**Affiliations:** ^1^ School of Medicine Iran University of Medical Sciences Tehran Iran; ^2^ Department of Dermatology, Hazrat Fatemeh Hospital, School of Medicine Iran University of Medical Sciences Tehran Iran; ^3^ Department of Medicine, NY Health and Hospitals South Brooklyn Health Brooklyn New York USA; ^4^ Skin and Stem Cell Research Center Tehran University of Medical Sciences Tehran Iran

**Keywords:** erythema multiforme major, JAK inhibitors, Janus Kinase inhibitors, severe cutaneous adverse reaction, SJS, Systematic Review, TEN, TNF‐α inhibitor, Toxic epidermal necrolysis

## Abstract

**Background and Aims:**

Stevens‐Johnson Syndrome (SJS), toxic epidermal necrolysis (TEN), and erythema multiforme major (EM major) are severe dermatologic conditions characterized by varying degrees of skin detachment and involvement of mucosal membranes, often triggered by drug reactions or infections. The management of these conditions poses a significant challenge due to the potential for rapid progression to life‐threatening complications. Recently, emerging therapeutic approaches such as Tumor Necrosis Factor‐alpha (TNF‐α) blockers and Janus Kinase (JAK) inhibitors have shown promise in regulating the dysregulated immune response implicated in these disorders. A comprehensive literature review was conducted to evaluate the efficacy and safety of TNF‐α blockers and JAK inhibitors in treating SJS, TEN, and EM.

**Methods:**

A systematic review was conducted according to PRISMA guidelines. A comprehensive literature search was performed in PubMed, Scopus, Embase, Web of Science, and Google Scholar, up to September 5, 2025, on JAK inhibitors and TNF inhibitors in treating SJS, TEN, or EM major.

**Results:**

The current review studies 82 articles (748 patients), including case reports, cohort studies, and clinical trials. Various JAK inhibitors and TNF inhibitors, such as tofacitinib, etanercept, adalimumab, and infliximab, were assessed regarding effectiveness in treating SJS/TEN and EM. Notably, some cases demonstrated a rapid response to these inhibitors, resulting in faster re‐epithelialization and a reduction in the severity of lesions.

**Conclusion:**

TNF‐α blockers like infliximab and etanercept show promise for patients unresponsive to standard treatments, shortening acute phases, reducing hospitalization, and mortality rates, and enhancing healing. Further research, especially registry‐based studies, is crucial. Healthcare practitioners are advised to exercise caution and reserve novel drugs for managing hypersensitivity reactions.

## Summary

- **Steven Johnson Syndrome (SJS) and Toxic Epidermal Necrolysis (TEN):**
- Life‐threatening dermatologic emergencies.
- Characterized by extensive epidermal necrolysis and shedding.
- Result from T‐cell‐related immune system overactivation and excess cytokines like IL‐6, IL‐15, TNF‐α. This study summarizes findings from 82 articles with a total of 748 patients on the therapeutic regimens used in patients with SCARs, such as SJS, TEN, and EM major. Two main groups of drugs were examined: JAK inhibitors and TNF‐α blockers.
- **TNF‐α Blockers:**
- Drugs like infliximab, adalimumab, etanercept.
- Inhibit TNF‐α, associated with apoptosis and cytotoxicity.
- Strongly expressed in TEN lesions.
- **JAK Inhibitors:**
- Inhibit JAK1, JAK2, JAK3, tyrosine kinase enzymes.
- Target dysregulated JAK‐STAT pathways.
- Offer therapeutic potential in reducing inflammation and severity of SJS.
- Used in diseases like rheumatoid arthritis, and myelofibrosis.
- **Etanercept in TEN:**
- Added to corticosteroids in resistant cases.
- Shortens acute stage, re‐epithelialization time, and length of stay.
- Lowers mortality rates when administered promptly.
- Reduces initial corticosteroid need and complications, but may increase infectious events.
- **Adalimumab in TEN/SJS:**
- Used in resistant cases, even with delayed administration.
- Shortens re‐epithelialization time.
- Promising results in combination therapy.
- **Infliximab in TEN:**
- Single dose or added to other immunosuppressants.
- Controls mucocutaneous eruptions, even in critical cases.
- Risk of infectious events, but most patients benefit without side effects.
- **JAK Inhibitors in TEN:**
- Limited studies, but promising results.
- **Ruxolitinib** was successfully used in overlap cases.
- **Treatment for Recurrent Erythema Multiforme:**
- Maintenance therapy with JAK inhibitors like Tofacitinib, Lenalidomide, and Thalidomide.
- Adalimumab as first‐line treatment, leading to complete remission.
- Reported adverse reactions include neuropathy and asthenia.

**Abbreviations**
BSA: body surface area
IVIG: intravenous immunoglobin
JAK: Janus kinas
RA: rheumatoid arthritis
SCARS: severe cutaneous adverse reactions
SJS: Steven Johnson Syndrome
TEN: toxic epidermal necrolysis
TNF: tumor necrosis factor
TYK2: tyrosine kinase 2

## 1 Introduction

Stevens ‐ Johnson syndrome (SJS) and Toxic Epidermal Necrolysis (TEN) are potentially life‐threatening dermatologic emergencies characterized by extensive epidermal necrolysis and shedding [1]. Such conditions generally occur 4–28 days after exposure to certain medications or viruses [2]. They are considered the same in terms of pathophysiology and different in terms of involvement of body surface area (BSA). The involved BSA in SJS and TEN are below 10% and over 30%, respectively. A 10%–30% BSA involvement is known as SJS/TEN overlap [1]. Another differential diagnosis of the SJS/TEN spectrum is the erythema multiforme (EM) which is usually a mild reaction to certain medications or viruses such as herpes simplex virus [3]. EM in most cases is controlled by supportive care and topical treatments [2]. However, EM has a severe form named EM major which resembles SJS and TEN in severity and clinical progression.

The current therapeutic approach consists of immediate cessation of causative medication, supporting treatments, wound care, and controversial medical agents which could be used to cease these reaction pathways [2, 4]. Currently, systemic corticosteroids, intravenous immunoglobulins (IVIGs), and other immunomodulators including cyclosporine are the most common medications used for TEN/SJS and EM major. There are also other classes of medications that have been used in solo or combination in some cases [5, 6]. Due to the severity, unpredictable course, and high mortality rate, many patients with such conditions need immediate admission to intensive care units for multidisciplinary care.

Despite the widespread use of systemic glucocorticoids, cyclosporine, and intravenous immunoglobulin (IVIG) in the management of SJS/TEN and EM major, the evidence supporting these therapies remains heterogeneous and, in some cases, controversial [4]. Their clinical use may also be limited by variable efficacy, potential adverse effects such as secondary infections, nephrotoxicity, and other treatment‐related complications, as well as financial burden and restricted accessibility in some healthcare settings [5, 6]. Given these limitations and the immune‐mediated pathophysiology of SCARs, emerging targeted therapies such as TNF‐α blockers and JAK inhibitors have gained increasing attention. Therefore, a comprehensive systematic review of these newer immunomodulatory agents is needed to summarize the available evidence and evaluate their potential role in addressing the shortcomings of conventional treatment approaches.

The main histopathologic features of the TEN/SJS spectrum include the separation of dermal and epidermal layers, necrosis of the epidermis, and apoptosis of keratinocytes. These changes are the result of immune system over‐activation through different T‐cell‐related immunologic pathways [7, 8]; cytokines such as IL‐6, IL‐15, TNF‐α, and so forth are elevated both in the mucocutaneous bullae and serum of patients with TEN/SJS [9]. Meanwhile, some studies demonstrated a positive correlation between a greater amount of cytokines like IL‐15 and the severity of the disease [10]. Considering the pathophysiology, certain types of medications could be used to control the progression of such conditions; two therapeutic target groups are the Janus kinase (JAK) family and tumor necrosis factor (TNF).

JAK family consists of JAK1, JAK2, JAK3, and Tyrosine kinase 2 (TYK2) enzymes, involved in the JAK/signal transducer and activator of transcription proteins (JAK‐STAT) pathway in lymphocytes. Consequently, JAK inhibitors can halt the JAK‐STAT pathway and lymphocyte activity [10]. This effect prompted studies exploring JAK inhibitors as therapeutic options for immune‐mediated conditions such as myelofibrosis and rheumatoid arthritis (RA) [10, 11].

On the other hand, TNF, formerly known as TNF‐α, is a cytokine involved in cell death by direct cytotoxicity and induction of apoptosis [12]. Inhibition of TNF is an effective treatment in non‐infectious inflammatory conditions, such as Crohn's disease, RA, and psoriasis [13]. Strong TNF expression in TEN lesions has encouraged studying TNF inhibitors as potentially effective treatments and resulted in promising outcomes [12, 14, 15].

In the end, due to the potentially life‐threatening manifestations of SJS and TEN, immune‐mediated pathophysiology, and a growing use of JAK and TNF inhibitors in immune‐mediated conditions, we conducted a comprehensive systematic review of JAK/TNF inhibitors in the treatment of TEN/SJS, or EM major. This review intends to summarize the available evidence and support clinical decision‐making.

## 2 Methods and Materials

The current systematic review was performed according to the PRISMA (Preferred Reporting Items for Systematic Reviews and Meta‐Analyses) statement; eligible cases were identified by the population‐intervention‐comparator‐outcomes‐study design (PICOS) framework: Population, humans experiencing hypersensitivity reactions of TEN, SJS, and severe cases of EM. Intervention, using any medications in the class of JAK inhibitors or TNF blockers to control cutaneous manifestations of those conditions. Comparator, patients with the same hypersensitivity reactions treated with conventional therapeutic regimens such as corticosteroids, IVIG, or other immunomodulator medications. Outcomes, any differences in the time of hospitalization/re‐epithelialization, side effects, complications, and mortality rate, and so forth.

### 2.1 Search Strategy

**Figure 1:**
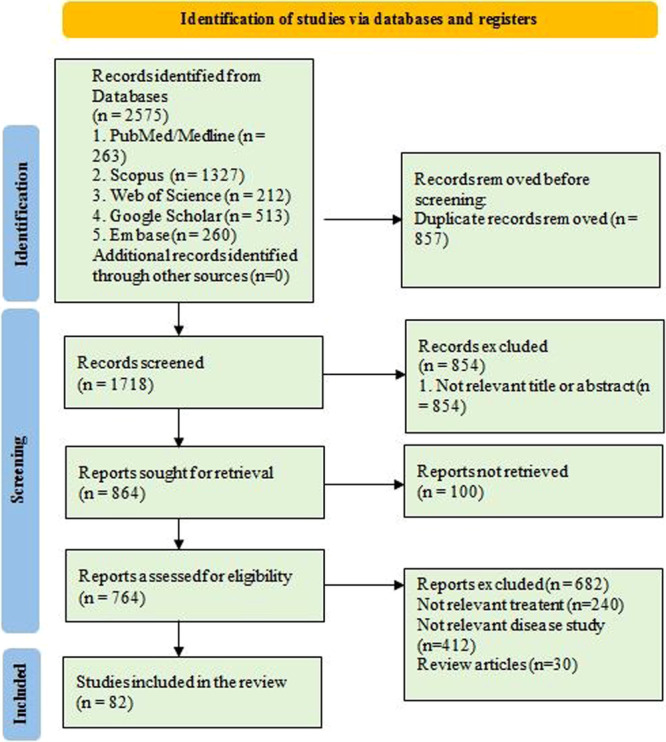
PRISMA 2020 flow diagram for systematic review.

A systematic review was conducted according to PRISMA guidelines (Figure 1). A comprehensive literature search was performed in PubMed, Scopus, Embase, Web of Science, and Google Scholar, up to September 5, 2025.

The search strategy was developed using combinations of keywords and Boolean operators (AND, OR). The main search terms included “Stevens‐Johnson syndrome,” “toxic epidermal necrolysis,” “erythema multiforme major,” “TNF inhibitors,” “etanercept,” “adalimumab,” “infliximab,” “JAK inhibitors,” and “tofacitinib.” These terms were combined using appropriate Boolean operators to optimize the search strategy (Table 1).

**Table 1:** Search strategies for databases.

PubMed	(“Toxic epidermal necrolysis “ OR “TEN” OR “ Stevens‐Johnson syndrome “ OR “SJS” OR “ TEN/SJS “ OR “Erythema multiforme major” OR “ Erythema multiforme”) AND (“TNF‐α blockers “ OR “ TNF‐α inhibitors” OR “ JAK inhibitors “ OR “ Tyrosine kinase 2 inhibitors “ OR “ TYK 2 inhibitors “ OR “Adalimumab” OR “ Etanercept” OR “ Infliximab” OR “certolizumab pegol” OR “golimumab” OR “tofacitinib” OR “baricitinib” OR “ruxolitinib” OR “upadacitinib” OR “fedratinib” OR “abrocitinib” OR “ritlecitinib” OR “deucravacitinib”)
Scopus	(TITLE‐ABS‐KEY(“Toxic epidermal necrolysis “ OR “TEN” OR “ Stevens‐Johnson syndrome “ OR “SJS” OR “ TEN/SJS “ OR “Erythema multiforme major” OR “ Erythema multiforme”)ANDTITLE‐ABS‐KEY(“TNF‐α blockers “ OR “ TNF‐α inhibitors” OR “ JAK inhibitors “ OR “ Tyrosine kinase 2 inhibitors “ OR “ TYK 2 inhibitors “ OR “Adalimumab” OR “ Etanercept” OR “ Infliximab” OR “certolizumab pegol” OR “golimumab” OR “tofacitinib” OR “baricitinib” OR “ruxolitinib” OR “upadacitinib” OR “fedratinib” OR “abrocitinib” OR “ritlecitinib” OR “deucravacitinib”))
Web of Science	TS = (“Toxic epidermal necrolysis “ OR “TEN” OR “ Stevens‐Johnson syndrome “ OR “SJS” OR “ TEN/SJS “ OR “Erythema multiforme major” OR “ Erythema multiforme”) AND TS = (“TNF‐α blockers “ OR “ TNF‐α inhibitors” OR “ JAK inhibitors “ OR “ Tyrosine kinase 2 inhibitors “ OR “ TYK 2 inhibitors “ OR “Adalimumab” OR “ Etanercept” OR “ Infliximab” OR “certolizumab pegol” OR “golimumab” OR “tofacitinib” OR “baricitinib” OR “ruxolitinib” OR “upadacitinib” OR “fedratinib” OR “abrocitinib” OR “ritlecitinib” OR “deucravacitinib”)

Filters were applied where applicable, including limiting results to human studies and English‐language publications.

### 2.2 Literature Screening

For the screening process, EndNote software, version 20, was utilized. Before screening 857 duplicates were detected by EndNote and removed. During the screening, two investigators reviewed all the literature by reading titles and abstracts to ensure their quality was included in data extraction, and the remaining duplicates were removed manually. Disagreements were resolved with a group discussion or the consensus of the corresponding investigator. In the last phase of screening, full texts were reviewed by three investigators, and 82 publications were selected for data extraction. In addition to database searching, the reference lists of included studies were manually screened to identify any additional relevant articles.

### 2.3 Inclusion and Exclusion Criteria

The inclusion criteria comprised English articles about the effect of JAKi or TNF‐α blocker medications on mucocutaneous manifestations of TEN/SJS or EM major. Exclusion criteria were reviews, investigational studies (animal, in‐vitro/ex‐vivo), treating TEN/SJS and EM major with medications other than JAK inhibitors or TNF‐α blockers, and studies considering the use of these medications in other medical conditions.

### 2.4 Data Extraction

Extracted data include (i) study characteristics: name of the authors, year, design of the study, county (ii) patients’ characteristics: age, gender, sample size, past medical history, the severity of the reactions and the involvement percentage, (iii) details on the treatment: duration between the onset of lesions and first dose of JAKi/TNF blockers, other treatments before or alongside JAKi/TNF blockers, duration of treatment, route of administration, and number of doses, and (IV) results: duration of hospitalization, side effects, complications, time to re‐epithelialization, and possible relapses. Microsoft Word software, version 16.56, was utilized for data extraction. Three investigators were involved in the data collection process, independently. Some main features of extracted data have been summarized in Tables 2, 3, 4, 5, 6, 7.

**Table 2:** Summary of case reports/series on etanercept therapy of SCARS.

Author, year	Sex, age	Severity and involvement	Comorbidity	Other treatment	Time to treat (days)	Side effects	Re‐epithelialization time (days)
Natsis, 2022	F, 20	60%	NA	NA	NA	NA	30
Ridha, 2021	M, 76	54%	HTN, Hailey Hailey, RCC & nephrectomy	No	1	NA	4
Faris, 2021	F, 71	90	NA	No	9	SIRS, AKI, electrolyte imbalance	Death despite resolutions of cutaneous manifestations
	F, 74	> 90	NA	Prior cyclosporine	20	AF, bleeding	Death despite resolutions of cutaneous manifestations
	F, 40	> 40%	NA	Prednisolone, cyclosporine	3	arrest	Death despite resolutions of cutaneous manifestations
Famularo, 2007	M, 59	60%	HTN, IHD, CKD	Prior and alongside prednisolone and cyclosporine	4	died of DIC at day 10	death despite complete skin healing on day 6
Sadighha, 2009	F, 56	NA	NA	Prednisolone	1	NA	18
Didona, 2015	NA	SCORTEN 2‐6	NA	No	NA	No	7–20 (median 8.5)
Paradisi, 2014	6 F, 4 M/61.6	SCORTEN 6	Cerebral neoplasm	No	< = 3	No	12
		SCORTEN 3	Bronchopneumonia	No	< = 3	No	8
		SCORTEN 2	Systematic lupus erythematous	No	< = 3	No	8
		SCORTEN 3	Pemphigus vulgaris	No	< = 3	No	12
		SCORTEN 4	Bronchopneumonia	No	< = 3	No	8
		SCORTEN 5	Cerebral neoplasm	No	< = 3	No	7
		SCORTEN 2	No	No	< = 3	No	8
		SCORTEN 6	Cerebral metastasis of breast cancer	No	< = 3	No	20
		SCORTEN 2	ICH	No	< = 3	No	9
		SCORTEN 3	Periarthritis	No	< = 3	No	9
Yardanova, 2022	F, 59	NA	Malignant melanoma	Prior IVIG, prednisolone	NA	No	NA
Paradisi, 2020	NA	Lowest SCORTEN:2, 5 patients with SCORTEN > = 5	NA	No	NA	No	2 deaths due to other causes, 15 prompt response versus 10/17 expected mortality rate
Gavigan, 2018	F, 11	25%	NA	Prior Methylprednisolone followed by cyclosporine	2	NA	NA
Li, 2020	F, 25	Almost 100%	NA	IVIG, methylprednisolone	0	NA	NA
de Perosanz, 2021	F, 78	15%–20%	Hypothyroidism/Dyspepsia/idiopathic pulmonary fibrosis	No	0	No	3 months to complete resolution
Singh, 2022	F, 47	25%	differentiated carcinoma of urothelial origin	Prior IVIG treatments	3	NA	5 days
Wang, 2019	F, 33	20%	acute upper respiratory tract infection	Prior and alongside Methylprednisolone, IVIG	5	No	15 days
Gao, 2020	M, 60	> 30%, SCORTEN 3	HTN/ocular lymphedema	Prior Methylprednisolone and IVIG	7	No	21 days
Didona, 2016	NA, 60	≥ 30%, SCORTEN 3	Pemphigus vulgaris	No	0	No	NA
Andrades, 2021	F, 57	NA	No	Combination therapy with IVIG, methylprednisolone and cyclosporine	NA	No	a few weeks
Osawa, 2022	M, 70	SCORTEN 4	metastatic prostate cancer	Prior IVIG and methylprednisolone	5	No	42 days
Bakir, 2021	F, 49	> 30%, SCORTEN 2	NA	No	0	No	22 days
Gubinelli, 2009	F, 59	> 30%	Brain tumor/epilepsy	Prior methylprednisolone	10	No	20 days
Napolitano, 2013	F, 67.3	> 30%	case 1&2: Systemic lupus erythematous	Case 3: methylprednisolone	0	No	8–12 days
Choi, 2022	M, 46	> 90%, SCORTEN 3	ascending aortic dissection	Prior methylprednisolone and alongside dexamethasone	1	No	23 days
Chahal, 2018	F, 19	30%, SCORTEN 1	No	No	0	No	8 days
Lee, 2013	M, 32	40%, SCORTEN 1	HIV	Alongside methylprednisolone	1	No	14 days
Zander, 2021	M, 13	90%	No	Prior Methylprednisolone	4	No	13 days
Vural, 2013	F, 89	NA	Psoriasis	No	0	No	NA
Seminari‐ Vidal, 2017	NA	NA	NA	Prior corticosteroids, IVIG	NA	NA	NA
Nikitina, 2023	F, 50	< 10% SCORTEN 1□ 2	NA	Prior and alongside methylprednisolone	10	No	5 days
Estebanez, 2020	M, 8	30%,	Post‐traumatic stress disorder	IVIG, Corticosteroids	3	NA	16 days
Ghai, 2023	M, 44	> 50%	Diabetes mellitus type 2	Prior methylprednisolone	NA	NA	Death due to multiorgan failure
Eliades, 2020	M, 4	80%	NA	No	4	NA	16
	F, 7	80%	NA	No	4	NA	13
	F, 18	30%	NA	No	1	NA	Disease progress was halted before any denudation Occurred
	F, 7	50%	NA	No	1	NA	10
Chong, 2017	M, 77	SCORTEN 2	DM, HTN, CAD, Hyperlipidemia,	No	0	NA	5

Abbreviations: AF, atrial fibrillation; AKI, acute kidney injury; CAD, coronary artery disease; CKD, chronic kidney disease; DIC, disseminated intravascular coagulation; DM, diabetes mellitus; EM, erythema multiforme; HIV, human immunodeficiency virus; HTN, hypertension; ICH, intracranial hemorrhage; ICU, intensive care unit; IHD, ischemic heart disease; IVIG, intravenous immunoglobulin; LOS, length of stay; PIH, post‐inflammatory hyperpigmentation; RCC, renal cell carcinoma; SIRS, systemic inflammatory response syndrome; SJS, Stevens‐Johnson syndrome; TEN, toxic epidermal necrolysis.

**Table 3:** Summary of interventional studies on etanercept therapy.

Author, year	N of cases	Sex, age	SCORTEN/involvement	Time to treat	Case results	Control treatment	Control results	Other results
Didona, 2019	30	NA, 18‐90	NA	NA	2 patients died of other causes Observed death: 2 Expected: 10	—	—	Median 8.5 days re‐epithelialization, the comparison between observed versus expected deaths was statistically significant (*p* = 0.012)
Sivagnanalingam, 2022	24(8 cases)	NA, NA	NA	NA	SMR 0.00	NA	SMR 0.56	LOS in SJS and overlap patients differed significantly between the 2 groups
C.H. Pham, 2019	13	38.5% M, 44.8 ± 21.1	Case: 3 Control: 2	Case: 5.2 Control: 2.7	SMR: 0.44 Mortality 15.4%, ICU days: 6.9, LOS: 9.8, infectious: 38.5%	NA	Mortality 10%, ICU days: 15.1, LOS: 16.4, infectious:57.5%	no significant difference in mortality, ICU days, length‐of‐stay or infections.
Tian, 2022	25 (14 cases)	NA, 11‐77	NA	NA	LOS 13.5 days	IVIG and corticosteroids only	LOS 19.0 days	Shorter hospitalization and less exposure to corticosteroids in combination therapy
Dreyer, 2021	22 (13 cases)	NA, NA	Case: 2.15 (30%) Control: 2.44 (23%)	NA	No mortality, re‐epithelialization days 8.1 days	IVIG	3 mortalities	treatment with etanercept may be less effective if IVIG and/or steroids are given prior to etanercept compared to when etanercept is given quickly
Worswick, 2020	30(21 cases)	NA, NA	NA	NA	Expected mortality 17.5% Actual mortality: 0%	Either supportive care or IVIG	Expected morality 20.8 versus actual mortality 30.3%	Lower SMR and actual mortality rate and relatively rapid response in etanercept group
Pham, 2018	13 cases	NA, NA	Case: 3 (54.3%) Control: 2 (46.3%)	5.7 days (vs. 2.6)	ICU days: 6.9, LOS:9.8 Infection: 38.5% Mortality: 15.4%	NA	ICU days: 15.1 LOS: 16.4 Infection: 57.5% Mortality: 10%	fewer ICU days, shorter total LOS and fewer infectious complications. There was no significant difference in mortality rate
S. T. Ao, 2022	25(15 cases)	56% M, 40.20 ± 18.28	NA	NA	Re‐epithelialization: 12 LOS: 12	Only methylprednisolone	Re‐ epithelialization:16 LOS: 14	the combination therapy significantly shortened the course of the initial steroid treatment and the duration of the acute stage, hospitalization stay, and skin re‐epithelialization.
Wang, 2018	96	NA	NA	NA	Predicted mortality: 17.7% Mortality: 8.3% Median skin healing time: 14 days Gastrointestinal bleeding: 2.6%	Traditional corticosteroids	Median skin healing time: 19 days Gastrointestinal bleeding: 18.2%	Decreased SCORTEN‐based mortality rate, skin healing time, GI bleeding) and TNF‐α and granulising secretions as mortality predictors in blister fluids and plasma of the etanercept group
Oginezawa, 2023	4	NA	Mean SCORTEN 2	NA	1 patient emerged sepsis	No control groups. Etanercept combination therapy was tested on priorly corticosteroid resistant patients	—	Recovery: 3.5 days, Re‐epithelialization: 18.5 days
Zhang, 2022	242	NA	NA	NA	Mortality: 0% SMR: 0 Median skin healing time: 12 (8.5–14) Lower adverse events of gastrointestinal bleeding	Corticosteroids monotherapy	Mortality: 6.63% SMR: 0.71 Median skin healing time: 13 [10, 11, 12, 13, 14, 15, 16, 17, 18]	Etanercept combination therapy reduces SMR, actual mortality rate, and skin healing time as well as adverse events such as gastrointestinal bleeding
						Corticosteroids+ IVIG	Mortality: 4.76% SMR: 0.30 Median skin healing time: 13.5 (10–19.5)	

Abbreviations: EM, erythema multiforme; ICU, intensive care unit; IVIG, Intravenous immunoglobulin; LOS, length of stay; SJS, Stevens‐Johnson syndrome; SMR, Standardized mortality ratio; TEN, toxic epidermal necrolysis.

**Table 4:** Efficacy and safety of etanercept for erythema multiforme major treatment.

Author, year	Sex, age	Severity and involvement	Comorbidity	Other treatment	Time to treat	Side effects	Re‐epithelialization time (days)
Trujillo‐Trujillo, 2018	M, 45	NA	No	Prior methylprednisolone	5 days	NA	2 days
Ling, 2017	F, 73	80%, SCORTEN 2	Hypertension, gastric ulcer	No	NA	No	14 days
Huang, 2023	F, 5	NA	No	No	3 days	No	NA

**Table 5:** Efficacy and study characteristics data on monotherapy with infliximab.

References/Year	Pt	Age (Mean)/Sex	Comorbidities	Prior treatments	Diagnosis	SCORTEN/BSA	Time to treatment	Outcome/Side Effects	Time to re‐epithelialization
Hunger 2005 [12]	1	69/F	HTN, Psoriasis, Hypothyroidism	No	TEN	2/ > 30%	3 days	Complete recovery	5 days
Wojtkiewicz 2008 [4]	1	17/F	No	Dexamethasone, IVIG	TEN	NA/80%	9 days	Complete Recovery & post‐inflammatory hyperpigmentation (PIH)	12 days
Scott‐Lang 2011 [6]	1	7/M	Autism, Epilepsy	IVIG	TEN	NA	2	Complete Recovery	7 days
Worsnop 2012 [7]	1	32/F	No	IVIG	TEN	2/ > 30%	3 days	Complete Recovery & PIH	9 days
Wallenborn 2017 [9]	1	44/M	No	Prednisolone	TEN	2/80%	NA	Complete Recovery & PIH	28 days
Al‐Shouli, 2005	1	67/M	CHD, DM, gout, HTN, obesity, hyperlipidemia,	Prednisolone	TEN	NA/40‐50%	10	Complete recovery	‘10
Seminario‐ Vidal, 2017	1	NA	NA	High dose steroids	TEN/GVHD	NA	NA	Complete recovery	NA
Zarate, 2013	2	76/M	NA	No	TEN	2/100%	NA	Complete Recovery	9
		20/F	HIV	methylprednisolone	TEN	2/100%	NA	Complete Recovery	7
Chafranska 2019 [11]	1	7/M	No	No	TEN	NA/30%	5 days	Resolution in 1 month and PIH	3
Kreft 2010 [5]	1	31/M	Disk herniation	Prednisolone	TEN	NA/ > 50%	NA	Complete Recovery & PIH	35 days
Fischer 2002 [1]	1	56/F	HTN	No	TEN	NA/35%	4 days	Complete Recovery	Within 28 days

Abbreviations: AKI, acute kidney injury; CHD, Coronary Heart Disease; CKD, Chronic kidney disease; DM, Diabetes mellitus; EM, erythema multiforme; GVHD, Graft versus host disease; HIV, Human immunodeficiency virus; HTN, Hypertension; IHD, Ischemic heart disease; IVIG, Intravenous immunoglobulin; PIH, post‐inflammatory hyperpigmentation; SJS, Stevens‐Johnson syndrome; TEN, toxic epidermal necrolysis;

**Table 6:** Efficacy and study characteristics data on combination therapy with infliximab.

References/Year	Pt	Age (Mean)/Sex	Comorbidities	Diagnosis	SCORTEN/BSA	Other Therapeutic modalities	Time to Treatment (day)	Outcome/Side Effects	Time to Re‐epithelialization (day)
Meiss 2007 [3]	3	42 1M2F	No	Overlap TEN & acute generalized exanthematous pustulosis	NA	methylprednisolone	NA	Complete Recovery	6‐14 days
Kreft 2014 [8]	1	11/F	No	TEN	NA	IVIG	9 days	Large extent of re‐epithelialization	14 days
Jiang 2018 [10]	1	36/F	Vogt‐Koyanagi‐Harada syndrome & secondary glaucoma	TEN	35%	methylprednisolone	5 days	Complete Recovery	7 days
Plant 2020 [12]	1	32/F	Alopecia totalis	TEN	NA	IVIG	NA	resolution of long‐term complications as well as Eruptions	NA
Zarate, 2013	2	51/F	NA	TEN	4/100%	methylprednisolone	NA	Recovery despite PTE, bacteremia	7
		17/F	epilepsy	TEN	3/50%	IVIG	NA	Recovery despite bacteremia	16
Gaitanis, 2012	3	Mean 72.6/M	HTN, AF, DM, BPH, and heart failure in cases	TEN	4/20% 3/40% 4/30%	High‐dose IVIG and corticosteroids	0	Recovery in two cases, one case passed away due to sepsis and multi organ failure	21, 22 days

Abbreviations: AF, atrial fibrillation; BPH, Benign prostatic hyperplasia; CKD, chronic kidney disease; DM, diabetes mellitus; EM, erythema multiforme; HTN, hypertension; IHD, ischemic heart disease; IVIG, intravenous immunoglobulin; PIH, post‐inflammatory hyperpigmentation; SJS, Stevens‐Johnson syndrome; TEN, toxic epidermal necrolysis.

**Table 7:** Efficacy and safety of different therapeutic regimens on recurrent erythema multiforme cases.

Author, year	Comorbility	Drug and dosage	Prior treatments	Final result	Follow up duration	N
Konda, 2022	NA	Tofacitinib 5 mg BD	Corticosteroids, cyclosporine, dapsone, tacrolimus, anakinra, rituximab, adalimumab, apremilast, mycophenolate mofetil, and intravenous immunoglobulin (IVIG)	Complete remission without side effects	2 years	1
Csiky‐Sessoms, 2019	NA	Tofacitinib 5 mg BD	Systemic corticosteroids, mycophenolic acid, famciclovir, topical tacrolimus, thalidomide, apremilast and etanercept	lesions improved but relapsed after drug discontinuation	8 months	1
Damsky, 2016	papillary thyroid cancer in remission,	Tofacitinib 5 mg BD	Topical corticosteroids, dapsone, valacyclovir, cyclosporine, azathioprine, mycophenolate mofetil, or methotrexate did not permit tapering of prednisone to less than 20 mg/d without a flare up	Complete remission	8 months	1
Murphy, 2021	No	Tofacitinib 100 mg BD, upadacitinib 15 mg daily was added in one case	No	Complete remission with titration of tofacitinib in all of the cases	NA	4
Drahy, 2018	NA	Lenalidomide 20 mg daily	Systemic corticosteroids, valacyclovir, and thalidomide at low and high doses	Partial remission	5 months	3
		Lenalidomide 10 mg daily		Complete remission	1 month	
		Lenalidomide 10 mg daily		Complete remission	1 month	
Chen, 2008	NA	Thalidomide 100 mg daily	Oral corticosteroids and topical agents, empiric valacyclovir	Complete remission	2 weeks	1
Moisson, 1992	Reiter syndrome in one of the cases	Thalidomide 100 mg daily	Steroid. Acyclovir, hydroxychloroquine, dapsone, high‐dose IVIG, colchicine and potassium iodide	Complete remission in one of the cases, and only effective on oral ulcers in the other case, Abnormality in EMG and neuropathy in both cases	6 months	2
Bahmer, 1982	NA	Thalidomide 200 mg then 100 mg daily	NA	Complete remission with 100 mg daily maintenance dose	NA	1
Cherouati, 1996	NA	Thalidomide 100 mg daily	Acyclovir, Prednisolone	Lesions disappeared within a week and remission was maintained with low dose‐treatment.	NA	26
Varma, 2006	Psoriasis	Thalidomide 100 mg daily	Dapsone	Psoriasis exacerbation	Treatment stopped within 2 weeks due to psoriasis exacerbation	1
Roux, 2021	NA	Thalidomide 50‐100 mg	Resistant to at least one drug (not mentioned)	Complete remission in 6 months in 2/3 of the cases despite asthenia (46%), and neuropathy (40%)	6 months	35
Baillis, 2017	NA	Adalimumab 40 mg/week	No	Complete remission	6 months	1

Abbreviations: EMG, Electromyography; IVIG, intravenous immunoglobulin.

### 2.5 Bias Assessment and Quality Evaluation

The NIH quality assessment tool was utilized for the bias assessment of case reports and case series [16]. The methodological quality and synthesis of the case reports and case series, suggested by Murad et al., 2018 [17] was also used for these studies. Newcastle‐Ottawa Quality assessment scale for non‐randomized studies as well as the Cochrane risk of bias tool was also used for other studies. The risk of bias assessment was conducted independently by multiple investigators. Disagreements were resolved through discussion and consensus, with involvement of a third reviewer when necessary.

### 2.6 Data Analysis

Results were systematically reviewed and described. Microsoft Word software, version 16.56, was used in the process.

Descriptive summary measures were calculated for outcomes that were reported with sufficient consistency across studies. For continuous outcomes such as re‐epithelialization time, crude averages were derived from the reported subgroup‐level means or medians. Formal pooled analyses for response rate and mortality were not performed across all TNF‐α blocker studies because of substantial heterogeneity in outcome definitions, denominators, and reporting formats.

Qualitative variables were summarized as frequencies and percentages. Quantitative variables were reported as means or medians according to the distribution characteristics and the reporting format of the original studies included in this review. Due to the heterogeneity of study designs, interventions, and outcome reporting, formal meta‐analysis was not feasible, and descriptive summary measures were used. Subgroup analyses were interpreted descriptively, and reported *p* values were considered within the methodological limitations and heterogeneity of the included studies.

## 3 Results

A total of 82 articles with a total number of 748 patients were included in this study. Among these, 64 were case reports/case series, 14 were cohort studies (11 retrospective cohorts and 3 prospective studies), 2 were parallel randomized control studies, and 2 were single‐arm trials. A summary of findings is provided here based on therapeutic regimens.

### 3.1 JAK Inhibitors

#### 3.1.1 Tofacitinib

Tofacitinib twice a day was successfully used to control some severe flare‐ups in two patients with chronic bullous EM as well as a herpes‐associated EM. This treatment controlled the flare‐ups for a long period; however, tapering the medications evolved into a flare‐up, suggesting the need for lifelong tofacitinib [18, 19, 20]. In one study, a combination of tofacitinib and upadacitinib was successfully treated with recurrent EM; a titration dose was necessary [21].

Tofacitinib was generally administered twice daily; however, reported dosing regimens varied across studies, with commonly used doses in other clinical settings ranging from 5 mg to 10 mg twice daily. In the included cases, exact dosing was not consistently reported, reflecting variability in clinical practice [18, 19, 20, 21].

#### 3.1.2 Ruxolitinib

There was a complicated SJS case in which intravenous ruxolitinib was started followed by corticosteroid therapy. The treatment failed due to general deterioration i.e. worsening liver failure, acute kidney injury (AKI), and coagulopathies [22].

An 11‐year‐old pediatric patient with refractory T‐cell lymphoma, after receiving peripheral blood stem cell therapy, showed mucocutaneous manifestation and more than 80% body area involvement. Administration of steroids and IVIG failed to control the condition. A single dose of ruxolitinib was administered on day 9 which reversed the disease progression, and subsequent complete resolution of lesions on day 17 [23].

### 3.2 TNF Blockers

#### 3.2.1 Etanercept

Etanercept has been used in many cases of TEN or SJS/TEN overlap syndrome (Table 2). In many cases, etanercept was tried due to failures in conventional therapies such as IVIG and corticosteroids. Mostly, a quick response to a single dose of etanercept was observed [24, 25, 26, 27, 28, 29, 30, 31, 32, 33, 34, 35, 36, 37, 38, 39, 40].

Etanercept was typically administered as a subcutaneous injection, most commonly at a dose of 50 mg per injection in reported cases, although dosing frequency and duration varied across studies [24, 25, 26, 27, 28, 29, 30, 31, 32, 33, 34, 35, 36, 37, 38, 39, 40].

A mean lag of 6.45 days between the onset of eruptions and etanercept therapy was reported and the average time for re‐epithelialization was 18 days. In some studies, patients received etanercept as first‐line therapy with a mean lag of 1.95 days of hypersensitivity reactions; complete resolution of lesions was reported after 14.15 days (mean) [41, 42, 43, 44, 45, 46, 47, 48, 49, 50]. The last group of patients were those who underwent a combined therapy of etanercept and other medications (i.e., corticosteroids, IVIG, and other immunomodulator agents); treatment was initiated within the first 3 days (mean: 1.33) and eruptions took an average of 14.16 days to re‐epithelialize [38, 51, 52, 53, 54, 55, 56]; two pediatric patients with 25% and 30% of body surface area involvement with a TEN‐like reaction showed improvement after a combination therapy of etanercept, systematic corticosteroids, IVIG, and cyclosporine [25, 57].

In an extreme case of a 25‐year‐old female with SJS, which was unresponsive to high doses of methylprednisolone and IVIG pulse, etanercept was initiated while the patient had almost 100% cutaneous involvement as well as severe agranulocytosis, thrombocytopenia, acute heart failure, pulmonary edema, and liver dysfunction. After 3 doses of etanercept, the lesions started to improve and a complete remission was reported within 4 months without any side effects. Also, a 13‐year‐old patient received etanercept after a 4‐day unsuccessful treatment with methylprednisolone and showed a complete resolution of eruptions in 13 days [34]. The reported cases were among the most severe reactions with up to 90% BSA involvement and a final resolution was achieved in many of them [31, 33].

Another notable report was a series of four pediatric patients with TEN, as young as 4 years old, initially managed with etanercept; in one of the patients, the initiation of etanercept therapy stopped the disease progression before the occurrence of histopathologic re‐epithelialization [58].

Despite successful therapeutic experiences, a case report in a patient who was under long‐term etanercept therapy for psoriasis flare‐ups showed no preventive effect with this medication and the patient eventually evolved TEN reaction [59].

Major complications were reported in two patients with several comorbidities and a TEN reaction involving more than 50% of their BSA who both eventually died of DIC and multi‐organ failure after adding etanercept to their prior corticosteroid regimens [32]. A single‐arm trial study evaluating the safety and efficacy of etanercept in 4 cases of SJS/TEN not responsive to steroids; despite the remarkable improvement, a patient developed sepsis that was hopefully cured with treatment. The mean time for re‐epithelialization was 13.5 days and the actual mortality rate was zero while a 12.1% mortality rate was predicted based on the patient's SCORTENs [37]. In a case series of three patients with TEN by Faris, all of the patients eventually passed away after adding etanercept to cyclosporine. The main reason for death was reported as electrolyte imbalance, AKI, and systemic inflammatory response syndrome (SIRS) in case one; atrial fibrillation (AF) and fetal bleeding in case two; cardiac arrest in case three. It was reported that adding etanercept to other immunosuppressives such as corticosteroids or cyclosporine may be associated with an increased risk of complications, including infections, although the available evidence remains limited and inconclusive [33].

Nine Cohort studies compared the effect of etanercept to conventional medications in treating hypersensitivity reactions; Details of these studies can be found in Table 3. Pham et al., 2019 showed that there is no significant difference in mortality rates, length of hospitalization (LOS), length of intensive care unit (ICU) admission, or infection rate between combined etanercept‐treated and non‐etanercept treated groups; however, the former group tended to experience a longer delay in response to the treatment [60]. Meanwhile, other studies showed a significant difference due to shorter LOS in the group of patients under etanercept therapy [61, 62, 63, 64, 65, 66]. Warwick et al., 2011 studied the effect of etanercept on 9 patients with SJS and compared the results to 21 patients receiving traditional treatments. The final report showed a lower mortality rate in etanercept group (0.00 vs. 30%) [67].

In another cohort study, Ao ST et al., 2022 concluded that compared to steroid monotherapy, combination therapy with etanercept significantly shortened the course of initial steroid therapy and the duration of the acute stage [66]. Dreyer et al., 2021 also reported that the most effective use of etanercept would be achieved when the treatment is started earlier compared to those receiving IVIG and/or steroids before etanercept [68]. Worswick et al., 2020 compared the mortality rates between patients treated with etanercept after failure of corticosteroid therapy with patients treated with etanercept as an initial therapy in a retrospective cohort study. Results showed a lower mortality rate in the first group (zero actual deaths vs 17.5% expected mortality rate in the second group and 20.8% vs 33% predicted mortality in the first group). Also, in another study by Didona et al., 2019 etanercept led to a significant reduction in actual mortality rate in TEN/SJS patients compared to their SCORTEN‐based expected mortality [61].

Wang et al., 2018 designed a randomized control trial on etanercept for patients with SJS/TEN spectrum in 2018 to compare etanercept with corticosteroid. 93 patients were enrolled in this study and were randomly allocated to either etanercept or corticosteroids group. Results of this study showed a decreased mortality rate based on the SCORTEN prediction model (8.3% vs. 17.7) and, a shorter healing time of the eruptions (median 14 days vs. 19 days in the corticosteroids group). Meanwhile, a lower rate of gastrointestinal hemorrhage as one of the disease complications was observed in the etanercept group (2.6% vs. 18.2% in the corticosteroids group) [69]. Regarding the safety and efficacy of etanercept plus corticosteroids compared to corticosteroid monotherapy, Zhang et al., 2022 conducted a comparative retrospective cohort. Results showed a reduction in the standard mortality rate and actual mortality rate as well as a shorter healing time of the lesions and lower rates of gastrointestinal adverse events in the combination group [65].

In a multi‐center single‐arm trial study designed by Oginezawa et al., 2023, etanercept was used in a group of patients resistant to corticosteroids. The patients in this trial had a mean recovery time of 3.5 days and a mean re‐epithelialization time of 13.5 days. One patient eventually expired due to severe sepsis despite being on etanercept treatment [37].

Besides SJS and TEN, Etanercept was also used in some cases of EM major (Table 4). In one study an EM major reaction developed after H. Pylori therapy in a 73‐year‐old woman. The initial SCORTEN was 2 with an 80% of body surface involvement. Due to active gastric ulcer and gastrointestinal bleeding, the use of corticosteroids was postponed and 6 doses of etanercept injection were used followed by some unsuccessful conservative therapies such as compound glycyrrhizin. A rapid response without any side effects was reported in this case and a complete remission of lesions was reported on day 14 [70]. Another case was a 45‐year‐old man (with no past medical history) with a severe form of EM who was unresponsive to methylprednisolone. Etanercept was added to the treatment regimen 5 days after symptoms onset; a complete epithelialization was reported 2 days after etanercept therapy [35]. Also, etanercept was tried as a first‐line therapy for a 5‐year‐old pediatric patient with an atypical form of EM major which resulted in a complete remission without any side effects [71].

For etanercept, the reported subgroup‐level re‐epithelialization times were 18 days in cases treated after failure of conventional therapies, 14.15 days in first‐line therapy studies, and 14.16 days in combination‐therapy studies, corresponding to a crude average of approximately 15.4 days across these summarized subgroups. In EM major cases treated with etanercept, re‐epithelialization time was reported in two cases (2 and 14 days), yielding a crude mean of 8 days. For infliximab, the average re‐epithelialization time reported in the included studies was 16.6 days for monotherapy and 14.4 days for combination therapy.

#### 3.2.2 Adalimumab

In a study, after noticing that a 6‐year‐old pediatric patient with SJS was unresponsive to a combined regimen of corticosteroids and human immunoglobin, two IV doses of adalimumab were added to the prior regimen. Despite the 11‐day lag between adalimumab therapy and the onset of rashes, a gradual resolution in cutaneous manifestations was observed [72].

Two other studies respectively reported 70‐ and 60‐year‐old patients with TEN, involving nearly 70% of their BSA, following the treatment for metastatic gastric malignancy and unresectable hepatocellular carcinoma. Both of the patients experienced disease progression despite the use of high‐dose systemic corticosteroids and IVIG treatment. The initial SCORTEN for one of these patients was calculated 5, further administration of adalimumab led to satisfactory results including faster lesion healing, reduction in duration of the treatment, and complete resolution of lesions by the end of hospitalization [73, 74].

In a retrospective chart review by Kherlopian et al., 2022 on SJS/TEN patients treated with adalimumab between 2017 and 2021 in Australia, all of these patients experienced a fairly prompt resolution of lesions without any adverse effects. Furthermore, they showed a shorter LOS (22.5 days vs 33 days in those who received non‐TNF‐α immunosuppressants such as glucocorticoids, IVIG, and cyclosporine) [75].

Furthermore, Gong et al., *2023* conducted two clinical cohorts on SJS/TEN to compare and analyze the efficacy and safety of adalimumab in combination with methylprednisolone versus monotherapy with methylprednisolone. This study showed that combination therapy remarkably shortened the time of mucocutaneous re‐epithelialization and healing compared to conventional therapy [76].

Adalimumab was also used to treat recurrent EM as a trial. The condition lasted for 2 years and was poorly controlled with daily doses of prednisolone. Scheduled doses of IV adalimumab (every other week) could suppress the active lesions and control the disease flare‐ups over time [77].

Adalimumab was administered as subcutaneous injections, typically following standard dosing regimens used in other inflammatory conditions (e.g., 40 mg), although variations were observed across studies [72, 73, 74, 75, 76, 77].

#### 3.2.3 Infliximab

Infliximab is a member TNF‐alpha blocker family. The efficacy and safety of infliximab in the treatment of TEN or SJS/TEN overlap syndrome have been investigated in several studies. The different characteristics of 17 case reports with a total of 23 participants are described in Tables 4 and 5. Each identified study was classified as either biologic monotherapy or combination therapy (Table 5). As shown in Table 4, in many of these studies monotherapy treatment with Infliximab was performed following the failure of primary therapeutic modalities (methylprednisolone, IVIG, and oral prednisolone) [12, 15, 40, 78, 79, 80, 81, 82, 83]. Other reports studied the efficacy and safety of infliximab in combination with other systemic therapies, as demonstrated in Table 6.

Infliximab was commonly administered as a single intravenous dose of 5 mg/kg, with some studies reporting repeated dosing depending on clinical response [12, 15, 40, 78, 79, 80, 81, 82, 83].

In 2002, the first case of effective treatment of TEN with Infliximab, with a re‐epithelialization duration of 4 weeks was reported; A rapid increase in the spread of the lesions ceased and no new lesions formed within the following days of the treatment initiation [84]. In two other studies, Hunger et al., 2005 and Al Shouli et al., 2005 reported that patients with TEN and other systemic comorbidities benefit from infliximab and most of the damaged skin was rapidly covered by a new epidermis [12, 85]. Additionally, Meiss et al., *2007* proved that infliximab can be an effective therapeutic measure in treating TEN overlapped with acute generalized exanthematous pustulosis [86].

In patients with a high percentage of skin involvement, BSA ≥ 80%, infliximab resulted in a satisfactory effect [15, 81]; however, there was a 9‐day lag between the onset of eruptions and infliximab therapy in one of the studies [15]. In infliximab monotherapy, the lag between the onset of eruptions and infliximab therapy was between 3 and 9 days in some cases [12, 15, 80, 87, 88].

Among pediatric participants, the effectiveness and rapid response of infliximab (less than 2 weeks) are reported [78, 87, 89]. Another pediatric case was reported by Lootah et al., 2023, about a 10‐year‐old pediatric patient with no known past medical history who was diagnosed with SJS with BSA > 90% and spared perineal region; after an unsuccessful course of IVIG and methylprednisolone pulse, infliximab was started and resulted in a rapid improvement and discharge after 5 days. In a follow‐up visit after 2 weeks, the primary skin lesions were fully resolved [90].

Most studies have indicated that a single dose of 5 mg/kg infliximab can result in a complete and irreversible recovery in both adults and children suffering from TEN; however, Jiang et al., 2018 reported a case in which the patient achieved a complete recovery with two doses of 4 mg/kg infliximab [88]. In most studies, re‐epithelialization of the skin occurred after 1–2 weeks, while in some cases it happened after 28–35 days and a complete resolution of eruptions was also achieved after a longer period [79, 83, 84, 91]. The average number of days for re‐epithelialization was 16.6 days in monotherapy and 14.4 days in the combination therapy group.

Regarding Side effects, Gaitanis et al., 2012 reported an elderly case suffering from multiple comorbidities such as AF, benign prostatic hyperplasia (BPH), hypertension (HTN), and heart failure who eventually passed away from sepsis and multi‐organs failure despite being on a therapeutic regimen of high‐dose corticosteroids, IVIG, and infliximab [92]. Bacteremia and pulmonary thromboembolism (PTE) were also reported in two cases by Zarate; however, they were managed successfully without any further complications [81].

*Paquet* et al., *2014* also designed a randomized controlled trial on 10 confirmed SJS patients who were randomly allocated to either N‐acetylcysteine monotherapy or N‐acetylcysteine plus infliximab therapy; a higher rate of mortality (2 cases, 40%) was observed in the combination therapy group compared to the predicted mortality rate based on SCORTEN (21.4%) and the mortality rate in the control group (20%). All of these mortalities were due to multi‐organ failure and septicemia [93]. However, these adverse events were not reported to be linked to medication administration.

### 3.3 Other Medications

#### 3.3.1 Thalidomide/Lenalidomide

Thalidomide and lenalidomide, although not classified as TNF‐α or JAK inhibitors, have been explored as immunomodulatory agents in selected cases and are therefore discussed as adjunctive therapeutic options.

Thalidomide is an immunomodulatory drug (ImiD) that is an effective treatment for EM; however, long‐term use is often limited due to adverse effects. Some studies have evaluated the effectiveness of ImiDs on EM (persistent and recurrent form) [94, 95, 96, 97, 98, 99, 100] (Table 7).

A retrospective cohort studied the treatment of thalidomide in 35 patients with chronic EM. The results were associated with complete remission after 6 months in two‐thirds of patients. The main adverse effects were asthenia and neuropathy, which led to the discontinuation of the treatment in some patients [94]. Neurological side effects of thalidomide in the treatment of EM were reported in other studies as well; Moisson et al., 1992, found that in combination therapy with thalidomide and other medications such as steroids, acyclovir, hydroxychloroquine, dapsone, high‐dose IVIG, and colchicine abnormality in EMG and neuropathy were detected. Additionally, therapy was effective only on the oral lesions in one of the patients [99].

The number of studies conducted on children was insufficient to prove the efficacy of thalidomide among the pediatric population, although Chen et al., 2008 has confirmed complete remission in a 15‐year‐old male patient with persistent EM [96]. Another study has suggested cautious use of thalidomide in patients with coexisting psoriasis. Thalidomide 100 mg daily was introduced for a 41‐year‐old woman and effectively controlled the EM eruptions, however, the exacerbation of psoriasis led to cessation of thalidomide after 2 weeks [97].

Recurrent EM was also successfully managed by thalidomide. In this condition, maintenance therapy with a lower dose of thalidomide was able to control flare‐ups without complications [98, 100].

Lenalidomide is a thalidomide analog which showed a better tolerability profile compared to thalidomide. Drahy et al., 2018, reported three female patients with chronic EM for 7–18 years with unsuccessful treatment with valacyclovir and thalidomide. For all three patients, treatment with lenalidomide was initiated at the dose of 10 mg/d for 21 days. One patient experienced a relapse, requiring dose adjustment which led to partial remission. Other patients showed complete remission after 1 month of treatment with no complications [95].

#### 3.3.2 Recombinant Medications

There were two studies to investigate the effect of recombinant human tumor necrosis factor‐α receptor: IgG Fc fusion protein in the treatment of TEN and SJS. A case report by Lu et al., 2020, showed successful treatment in an SJS patient resistant to glucocorticoids. Furthermore, another study on 22 patients with TEN revealed a successful response with no recurrence/complication, and eventually most skin lesions subsided after 2 weeks of therapy [101, 102].

## 4 Discussion

This study summarizes findings from 82 articles with a total of 748 patients on the therapeutic regimens used in patients with SCARs, such as SJS, TEN, and EM major. Two main groups of drugs were examined: JAK inhibitors and TNF‐α blockers.

The findings of this systematic review are consistent with previous reports suggesting a potential role for targeted immunomodulatory therapies, particularly TNF‐α inhibitors, in modifying the disease course of SCARs. Several studies have indicated that TNF‐α blockade may contribute to rapid cessation of disease progression and accelerated re‐epithelialization. However, the current body of evidence is largely derived from case reports, case series, and observational studies, which limits the strength of conclusions and highlights the need for well‐designed randomized controlled trials [24, 25, 26, 27, 28, 29, 30, 31, 32, 33, 34, 35, 36, 37, 38, 39, 40, 72].

Current clinical guidelines emphasize supportive care as the cornerstone of management for SJS/TEN, including fluid replacement, wound care, and infection control. The role of systemic therapies such as corticosteroids, cyclosporine, and intravenous immunoglobulin (IVIG) remains controversial due to inconsistent evidence regarding their efficacy and safety [1, 5, 23]. In this context, emerging therapies such as TNF‐α inhibitors and JAK inhibitors have attracted increasing attention as potential alternatives, although robust evidence supporting their routine use is still lacking.

Regarding JAK inhibitor drugs, tofacitinib was successfully used to control severe flare‐ups in two cases of chronic bullous EM and a herpes‐associated EM, but long‐term usage was required, as tapering the drug led to flare‐ups [12, 13, 14]. In contrast, intravenous ruxolitinib did not improve the general instability conditions in a complicated SJS case, and general deterioration continued even after the use of ruxolitinib, and finally, the patient passed away [22].

TNF‐α blockers were found to be more effective in treating SCARs. The most common drug among those considered in this review is etanercept which has been used in many cases of SJS/TEN spectrum. Etanercept has been used in cases where conventional therapies such as IVIG and corticosteroids have failed. In most cases, patients showed a rapid response to a single dose of etanercept, and a deterioration in cutaneous reactions was observed [24, 25, 26, 27, 28, 29, 30, 31, 32, 33, 34, 35, 57]. The average time for re‐epithelialization was 14.71 days. However, there were cases where etanercept was used as the first‐line treatment after hypersensitivity reactions, and the duration of the complete resolution was 16.58 days. There were also cases where etanercept was used in combination with other drugs (i.e., corticosteroids, IVIG, and other immunomodulator agents), and treatment was started within the first 2 days, taking an average of 15.40 days for eruptions to re‐epithelialize [41, 42, 43, 44, 45, 46, 47, 48, 49]. An adolescent case of 13 years old who received etanercept followed by a 4‐day unsuccessful treatment with methylprednisolone showed complete resolution of eruptions in 13 days [34].

The most severe reactions were reported in four cases with up to 90% BSA involvement, and all achieved final resolution [31, 33]. The only major side effect observed was in a patient with several comorbidities and a TEN involving 60% of BSA who eventually died of DIC after adding etanercept to his prior corticosteroids regimen [32]. Some temporary complications, such as electrolyte imbalance, Acute kidney injury, cardiac arrest, and septicemia in four cases were managed promptly, and all patients achieved complete re‐epithelialization under 30 days [33].

Eight cohort studies comparing etanercept to conventional regimens for hypersensitive reactions were included in the manuscript. Except for one of those which showed no significant difference in mortality rate, LOS, ICU admission, and infectious rate (despite the delayed onset of etanercept therapy), other studies indicated shorter hospitalization periods for the patients receiving etanercept [61, 62, 63, 64, 65].

Warwick's study on 9 SJS patients receiving etanercept showed a lower mortality rate compared to traditional treatments (0.00 vs. 30%) [67]. Ao.S.T.'s study concluded that combination therapy with etanercept significantly shortened the course of the initial steroid treatment and the duration of the acute stage compared to steroid monotherapy [66]. Dreyer's study showed that the most prominent effects of using etanercept would be observed in cases where treatment is started quickly, as opposed to those where IVIG and/or steroids are given before etanercept [68].

Adalimumab was also used in a pediatric patient with SJS, who was unresponsive to a combined regimen of corticosteroids and IVIG, and in a case of recurrent EM that had been poorly controlled with daily doses of prednisolone as a trial. In both cases, a gradual resolution in cutaneous manifestations was observed [73].

Several studies have investigated the effectiveness and safety of Infliximab, another TNF‐alpha blocker, in treating TEN or SJS/TEN overlap syndrome. Fifteen case‐report studies with a total of 21 participants were identified and classified as either biologic monotherapy or combination therapy. Monotherapy with Infliximab was performed in many of these studies after the failure of primary therapeutic modalities [12, 15, 40, 78, 79, 80, 81], and combination therapy was used in other studies. The average number of days to re‐epithelialization was 16.6 days and 14.4 days for monotherapy and combination therapy groups respectively [12, 15, 78, 80, 87, 89].

Among the reviewed articles, three studies mentioned the effectiveness and rapid response of this drug in less than 2 weeks in the treatment of pediatric participants [78, 87, 89]. In 2002, the first case of effective treatment of TEN with Infliximab, with a re‐epithelialization duration of 4 weeks, was reported [84]. Hunger et al., 2005 and Al Shouli et al., 2005 showed that patients with comorbidities also benefit from Infliximab [12, 85]. Meiss, 2007 showed its effectiveness in the overlap of TEN and acute generalized exanthematous pustulosis by reporting 3 cases [86]. Infliximab had a satisfactory effect on BSA ≥ 80% [15, 81], although there was a 9‐day lag between the onset of eruptions and Infliximab Therapy in some cases [15]. Re‐epithelialization occurred between 1 and 2 weeks in most studies, while some reported longer durations with dermatological follow‐up revealing the resolution of eruptions after a long time [79, 83, 84, 91].

Thalidomide and lenalidomide, although not classified as TNF‐α or JAK inhibitors, have been explored as immunomodulatory agents in selected cases and are therefore discussed as adjunctive therapeutic options.

Thalidomide, as another immunomodulatory drug, has shown efficacy in the treatment of EM, a hypersensitivity skin condition involving T‐cell and B‐cell responses, when antiviral therapy fails. The effectiveness of thalidomide on persistent and recurrent forms of EM has been evaluated in a limited number of articles [63], with a recent retrospective cohort study in 2019 on 35 patients reporting complete remission after 6 months in two‐thirds of patients, although some patients discontinued treatment due to adverse effects such as asthenia and neuropathy [63]. Neurological side effects have been reported in other studies when thalidomide was used in combination therapy with other drugs [68]. Studies in pediatric populations are insufficient to prove the efficacy of thalidomide, although a single study reported complete remission in a 15‐year‐old male [65]. Cautious use of thalidomide is recommended in patients with coexisting psoriasis [66].

Lenalidomide is a thalidomide analog and, as its alternative shows a better tolerability profile than thalidomide. Drahy et al., 2018 reported three women with chronic EM were treated with Lenalidomide 10 mg/d from Day 1 to Day 21 of a 28‐day cycle. One patient experienced a relapse, while the other two achieved complete remission after 1 month of treatment [64].

Two other studies found that 22 patients with TEN had an effective response to recombinant human tumor necrosis factor‐α receptor with most skin lesions subsiding after 2 weeks without recurrence or complications [101, 102].

From a clinical perspective, these findings suggest that TNF‐α inhibitors and JAK inhibitors may be considered as adjunctive therapeutic options, particularly in severe or refractory cases of SCARs. However, treatment decisions should be individualized, taking into account disease severity, comorbidities, and the potential risks associated with immunosuppression.

The heterogeneity of the included studies, including variations in study design, patient populations, treatment regimens, and outcome reporting, represents a major challenge in interpreting the available data. Additionally, the predominance of case‐based evidence introduces potential reporting and selection biases. Despite these limitations, a consistent trend toward favorable clinical outcomes, such as reduced disease progression and shorter re‐epithelialization time, was observed across multiple studies, suggesting a potential therapeutic benefit of targeted immunomodulation.

The methodological quality of the included studies varied depending on the study design. The majority of included studies were case reports and case series, which inherently have lower levels of evidence and are subject to reporting bias. Cohort studies demonstrated moderate quality, although most were retrospective in nature and susceptible to selection bias. The limited number of randomized controlled trials showed moderate methodological quality but were constrained by small sample sizes. Overall, heterogeneity in study design and methodological limitations should be considered when interpreting the findings (Table 8).

**Table 8:** Study‐by‐study draft quality assessment.

Author, year	Therapy	Study design	*N*	Assessment tool	Draft grade	Rationale
Natsis, 2022	Etanercept	Case report	1	NIH + Murad	Fair	Single‐patient report with outcome details; limited external validity and no comparator.
Ridha, 2021	Etanercept	Case report	1	NIH + Murad	Fair	Adequate clinical detail and short‐term outcome; single case and no control group.
Faris, 2021	Etanercept	Case series	3	NIH + Murad	Fair	Small series with clinically important outcomes; high risk of selection/reporting bias.
Famularo, 2007	Etanercept	Case report	1	NIH + Murad	Fair	Case adequately described but limited by single‐patient design and confounding co‐therapies.
Sadighha, 2009	Etanercept	Case report	1	NIH + Murad	Fair	Basic treatment‐outcome sequence reported; limited methodological depth.
Didona, 2015	Etanercept	Case series	NA	NIH + Murad	Fair	Series‐level outcome reporting present; details of patient selection and comparability limited.
Paradisi, 2014	Etanercept	Case series	10	NIH + Murad	Good	Relatively larger case series with multiple patient‐level outcomes; still uncontrolled.
Yardanova, 2022	Etanercept	Case report	1	NIH + Murad	Fair	Useful clinical description but single case with limited generalizability.
Paradisi, 2020	Etanercept	Case series	NA	NIH + Murad	Fair	Reports aggregated outcomes and expected‐vs‐observed mortality; still uncontrolled.
Gavigan, 2018	Etanercept	Case report	1	NIH + Murad	Fair	Pediatric case with treatment chronology; no comparator and limited follow‐up detail.
Didona, 2019	Etanercept	Retrospective cohort	30	Newcastle‐Ottawa Scale	Moderate	Comparative mortality analysis reported, but likely retrospective and prone to confounding.
Sivagnanalingam, 2022	Etanercept	Retrospective cohort	24	Newcastle‐Ottawa Scale	Moderate	Comparative data available, but sample remains limited and selection bias likely.
C.H. Pham, 2019	Etanercept	Retrospective cohort	13	Newcastle‐Ottawa Scale	Moderate	Comparator present and multiple outcomes assessed; small sample and treatment delay imbalance.
Tian, 2022	Etanercept	Retrospective cohort	25	Newcastle‐Ottawa Scale	Moderate	Comparative length‐of‐stay data available; likely retrospective and subject to confounding.
Dreyer, 2021	Etanercept	Retrospective cohort	22	Newcastle‐Ottawa Scale	Moderate	Comparator included and outcomes reported, but nonrandomized treatment allocation limits inference.
Worswick, 2020	Etanercept	Retrospective cohort	30	Newcastle‐Ottawa Scale	Moderate	Mortality comparison provided; residual confounding and case‐mix differences remain concerns.
Pham, 2018	Etanercept	Retrospective cohort	13	Newcastle‐Ottawa Scale	Moderate	Reports ICU/LOS/infection outcomes with controls, but very small sample.
S. T. Ao, 2022	Etanercept	Retrospective cohort	25	Newcastle‐Ottawa Scale	Moderate	Direct comparison to steroid monotherapy strengthens evidence, but nonrandomized design limits certainty.
Wang, 2018	Etanercept	Randomized controlled trial	96	Cochrane RoB	Moderate	Randomized design improves validity; still limited by sample size and possible trial‐level concerns.
Oginezawa, 2023	Etanercept	Single‐arm trial	4	NIH/descriptive assessment	Fair	Prospective treatment data but no control arm and very small sample.
Zhang, 2022	Etanercept	Retrospective cohort	242	Newcastle‐Ottawa Scale	High	Large comparative cohort with multiple outcomes; still observational with potential residual confounding.
Trujillo‐Trujillo, 2018	Etanercept (EM major)	Case report	1	NIH + Murad	Fair	Single severe EM case with response data; uncontrolled.
Ling, 2017	Etanercept (EM major)	Case report	1	NIH + Murad	Fair	Detailed response timeline reported, but single‐patient evidence only.
Huang, 2023	Etanercept (EM major)	Case report	1	NIH + Murad	Fair	Pediatric case with treatment chronology; limited external validity.
Hunger, 2005	Infliximab	Case report	1	NIH + Murad	Fair	Adequate clinical narrative and outcome, but single case.
Wojtkiewicz, 2008	Infliximab	Case report	1	NIH + Murad	Fair	Treatment and recovery described; no comparator.
Scott‐Lang, 2011	Infliximab	Case report	1	NIH + Murad	Fair	Pediatric case adds value but remains anecdotal evidence.
Worsnop, 2012	Infliximab	Case report	1	NIH + Murad	Fair	Reasonable case detail; limited generalizability.
Wallenborn, 2017	Infliximab	Case report	1	NIH + Murad	Fair	Documents recovery after prior therapy; confounding co‐treatment present.
Al‐Shouli, 2005	Infliximab	Case report	1	NIH + Murad	Fair	Case described with key outcomes; still uncontrolled.
Seminario‐Vidal, 2017	Infliximab	Case report	1	NIH + Murad	Poor	Important outcome reported, but substantial missing baseline and timing details.
Zarate, 2013	Infliximab	Case series	2	NIH + Murad	Fair	Small series with adverse events and recovery reported; no control arm.
Chafranska, 2019	Infliximab	Case report	1	NIH + Murad	Fair	Pediatric case with outcome details; limited methodological strength.
Kreft, 2010	Infliximab	Case report	1	NIH + Murad	Fair	Clinical response reported but uncontrolled and prone to publication bias.
Fischer, 2002	Infliximab	Case report	1	NIH + Murad	Fair	Historically important case; single‐patient evidence only.
Meiss, 2007	Infliximab	Case series	3	NIH + Murad	Fair	Small series with favorable outcomes; combination therapy complicates attribution.
Kreft, 2014	Infliximab	Case report	1	NIH + Murad	Fair	Clear treatment timeline with prior IVIG; no comparator.
Jiang, 2018	Infliximab	Case report	1	NIH + Murad	Fair	Adequate case‐level detail and recovery time; single case.
Plant, 2020	Infliximab	Case report	1	NIH + Murad	Poor	Outcome mentioned but incomplete quantitative detail limits appraisal.
Gaitanis, 2012	Infliximab	Case series	3	NIH + Murad	Fair	Series includes major adverse event data; very small and confounded by co‐therapies.
Konda, 2022	Tofacitinib	Case report	1	NIH + Murad	Fair	Good longitudinal follow‐up, but single case with extensive prior therapy.
Csiky‐Sessoms, 2019	Tofacitinib	Case report	1	NIH + Murad	Fair	Useful relapse information after discontinuation; anecdotal evidence.
Damsky, 2016	Tofacitinib	Case report	1	NIH + Murad	Fair	Detailed refractory disease history, but no comparator.
Murphy, 2021	Tofacitinib/Upadacitinib	Case series	4	NIH + Murad	Fair	Multi‐patient report with dose titration details; uncontrolled.
Drahy, 2018	Lenalidomide	Case series	3	NIH + Murad	Fair	Small series with response heterogeneity; no control group.
Chen, 2008	Thalidomide	Case report	1	NIH + Murad	Fair	Straightforward clinical response reported; single case.
Moisson, 1992	Thalidomide	Case series	2	NIH + Murad	Fair	Includes adverse effects and mixed efficacy, but very small and uncontrolled.
Bahmer, 1982	Thalidomide	Case report	1	NIH + Murad	Poor	Older report with limited methodological/reporting detail.
Cherouati, 1996	Thalidomide	Case series	26	NIH + Murad	Good	Larger case series with maintenance‐response data; still uncontrolled and vulnerable to bias.
Varma, 2006	Thalidomide	Case report	1	NIH + Murad	Fair	Captures clinically relevant adverse outcome; single‐patient evidence.
Roux, 2021	Thalidomide	Retrospective cohort	35	Newcastle‐Ottawa Scale	Moderate	Largest chronic EM dataset, but retrospective observational design limits causal inference.
Baillis, 2017	Adalimumab	Case report	1	NIH + Murad	Fair	Single recurrent EM case with follow‐up; uncontrolled.

## 5 Conclusion

Based on the available evidence, TNF‐α blockers and JAK inhibitors may represent promising therapeutic options, particularly in patients with severe or rapidly progressive SCARs, as well as in cases refractory to conventional treatments. These agents may be associated with faster disease control and improved clinical outcomes in selected patients. However, given the heterogeneity and limited quality of the available data, these findings should be interpreted with caution, and further high‐quality studies are required to better define the optimal target populations and treatment strategies.

## 6 Limitation and Recommendation

Several limitations should be considered when interpreting the findings of this study. First, the majority of included studies were case reports and case series, which represent a low level of evidence and are inherently subject to reporting and selection biases.

Second, the potential for publication bias cannot be excluded, as studies with positive outcomes are more likely to be published, particularly in the context of rare and severe conditions such as SCARs.

Third, substantial heterogeneity existed across studies in terms of patient populations, treatment regimens, and outcome definitions, which limits comparability.

Finally, a formal analysis of dose‐response relationships was not feasible due to inconsistent and incomplete reporting of dosing data across the included studies.

## Author Contributions

**Sadaf Salehi:** conceptualization, investigation. **Pooneh Torabi:** writing – original draft, methodology. **Mahsa Abbaspour:** validation, visualization. **Nima Hajizadeh:** writing – review and editing, software. **Sara Sadeghi:** writing – review and editing, writing – original draft. **Alireza Jafarzadeh:** supervision, project administration. **Azadeh Goodarzi:** supervision, project administration, visualization.

## Ethics Statement

An ethics statement is not applicable because this study is based exclusively on published literature.

## Conflicts of Interest

The authors declare no conflicts of interest.

## Transparency Statement

The Azadeh Goodarzi affirms that this manuscript is an honest, accurate, and transparent account of the study being reported; that no important aspects of the study have been omitted; and that any discrepancies from the study as planned (and, if relevant, registered) have been explained.

## Data Availability

All data produced in the present study are available upon reasonable request to the authors.
